# On the Surface Modification of LLZTO with LiF via a Gas-Phase Approach and the Characterization of the Interfaces of LiF with LLZTO as Well as PEO+LiTFSI

**DOI:** 10.3390/ma15196900

**Published:** 2022-10-05

**Authors:** Manuel Donzelli, Thimo Ferber, Vanita Vanita, Aamir Iqbal Waidha, Philipp Müller, Maximilian Mellin, René Hausbrand, Wolfram Jaegermann, Oliver Clemens

**Affiliations:** 1 Fachgebiet Materialdesign Durch Synthese, Fachbereich Materialwissenschaft, Technische Universität Darmstadt, Alarich-Weiss-Straße 2, 64287 Darmstadt, Germany; 2 Chemische Materialsynthese, Institut für Materialwissenschaft, Universität Stuttgart, Heisenbergstraße 3, 70569 Stuttgart, Germany; 3 Fachgebiet Oberflächenforschung, Fachbereich Materialwissenschaft, Technische Universität Darmstadt, Otto-Berndt-Straße 3, 64287 Darmstadt, Germany

**Keywords:** LLZTO, surface modification, interface stability, fluorination, XPS

## Abstract

In this study we present gas-phase fluorination as a method to create a thin LiF layer on Li_6.5_La_3_Zr_1.5_Ta_0.5_O_12_ (LLZTO). We compared these fluorinated films with LiF films produced by RF-magnetron sputtering, where we investigated the interface between the LLZTO and the deposited LiF showing no formation of a reaction layer. Furthermore, we investigated the ability of this LiF layer as a protection layer against Li_2_CO_3_ formation in ambient air. By this, we show that Li_2_CO_3_ formation is absent at the LLZTO surface after 24 h in ambient air, supporting the protective character of the formed LiF films, and hence potentially enhancing the handling of LLZTO in air for battery production. With respect to the use within hybrid electrolytes consisting of LLZTO and a mixture of polyethylene oxide (PEO) and lithium bis(trifluoromethanesulfonyl)imide (LiTFSI), we also investigated the interface between the formed LiF films and a mixture of PEO+LiTFSI by X-ray photoelectron spectroscopy (XPS), showing decomposition of the LiTFSI at the interface.

## 1. Introduction

Solid-state batteries are currently being investigated for their potential to be used together with metallic Li anodes to achieve high energy densities. They comprise the use of a solid electrolyte with high ionic conductivity and large electrochemical stability window. Li-conducting garnets such as Li_7_La_3_Zr_2_O_12_ (LLZO) [1] or Li_7−x_La_3_Zr_2−x_Ta_x_O_12_ (LLZTO) [2] can be stable towards metallic Li [3], and are of interest to be used in combination with ion-conducting polymers within hybrid electrolytes, e.g., consisting of polyethylene oxide (PEO), lithium bis(trifluoromethanesulfonyl)imide (LiTFSI), and LLZTO [4,5]. One intrinsic problem of garnet materials originates from their high basicity, and they can react with traces of water and CO_2_, forming Li_2_CO_3_ at the surface [6]. This can then act as a passivation layer, hindering the transport of Li ions between grains as well as the transfer to the active electrode materials [7]. In addition, the volume changes of this corrosion process are unfavourable, which can lead to ongoing degradation of the bulk material. Additionally, for hybrid electrolytes, the high basicity can result in reactions at the direct interface [8], and reducing the surface reactivity while maintaining a conductive surface is a challenge that needs to be overcome to successfully use this type of material.

Various methods have been proposed for the surface modification of garnet materials, ranging from dissolving LiOH/Li_2_CO_3_ via acid treatment [9,10] to the deposition of lithium-free salts as thin passivation layers, which, despite not being ion conductors themselves, do not block lithium ion transport (e.g. Al_2_O_3_ [11] or Sb_2_O_3_ [12]). Further, lithium halides (LiX, X = Cl, Br, I) [13] prepared by wet-chemical approaches have been reported to be functional coatings for garnets. Additionally, LiF, which shows high thermal and chemical stability, was considered recently and showed excellent coating properties, making garnet pellets wettable with metallic lithium [14]. However, the method suggested by the authors required the use of high vacuum deposition, making it difficult to be scaled up. Furthermore, melting LiF directly onto the surface of a garnet pellet was suggested [5]. While the authors could show a beneficial effect on Li_2_CO_3_ formation and no detrimental effect on the Li-ion diffusion, this technique requires temperatures above the melting point of LiF (T_melt_ = 845 °C).

For synthesis of garnet materials, the lithium precursor is normally used in excess to compensate lithium losses during material synthesis. This difficulty in balancing the Li to La/Zr concentration precisely must be considered as one origin for the existence of unfavourable Li-compounds at the surface. Transformation of this residue to LiF, as well as slightly degrading the garnet surface by using a mild gas-phase based fluorination, might therefore provide a promising route to functional and stable garnet surfaces.

Most fluorination agents are either highly corrosive (e. g. anhydrous HF) or can lead to extended material degradation. An alternative lies in providing HF in low concentrations by decomposition of fluorine-containing polymers such as PVDF [15], and this was used for topochemical fluorinations of powders or thin films, for the latter either by coating the polymer on top of the film or by heating it separately at ~200 °C, which causes the evaporation of shorter polymer molecules which can then decompose at the surface directly [16,17]. PVDF-based gas fluorination routes thus work by the partial release of HF species at the substrate surface.

Consequently, we suppose that this method could, in principle, function to transform surface-contaminating alkaline materials such as carbonates or hydroxides according to

OH^−^ + HF → H_2_O + F^−^ or CO_3_^2−^ + 2 HF → H_2_O + CO_2_ + 2 F^−^

while maintaining a low acidity via a low *p*(HF).

In this work, we report on the use of a gas-phase fluorination route for the surface protection of garnet-type LLZTO films with thin layers of LiF. We show that the method is suitable to protect thin films with flat surfaces from degradation to Li_2_CO_3_ under ambient conditions, while not reducing the conductivity significantly. In addition, we address the limitations of this method such as the gas-streaming profile in order to surface-protect porous geometries. In the second part, due to relevance of LiF coatings within composite electrolytes, we provide a detailed analysis of the interface between LLZTO and sputter-deposited LiF, as well as between LiF and stepwise deposited PEO/LiTFSI, complementary to our previous study of the interface between LLZTO and PEO/LiTFSI [8].

## 2. Experimental Section

### 2.1. LLZTO Thin Film Deposition

Li_6.5_La_3_Zr_1.5_Ta_0.5_O_12_ (LLZTO) thin films were deposited by LASER-assisted chemical vapor deposition (LA-CVD) on Si wafers (CrysTec GmbH, Berlin Germany) with an orientation of (100). For a detailed description of the deposition setup please see previous publications of our group [18]. As precursors, 2,2,6,6-tetramethyl-3,5-heptanedionato lithium (LiC_11_H_19_O_2_, STREM Chemicals GmbH, Kehl, Germany, 98%), lanthanum(III) acetylacetonate hydrate (La(C_5_H_7_O_2_)_3_·4H_2_O, abcr GmbH, Karlsruhe, Germany, 99.9%), tantalum(V) tetraethoxyacetylacetonate (TaC_13_H_27_O_6_, STREM Chemicals GmbH, Kehl, Germany, 99.99%), and zirconium(IV) acetylacetonate (Zr(C_5_H_7_O_2_)_4_, STREM Chemicals GmbH, Kehl, Germany, 98%) were mixed in stoichiometric ratio, with a 50 wt % excess of Li. As process gases, argon (1.45 slm (standard litres per minute), Air Liquide Deutschland GmbH, Düsseldorf, Germany, 99.999%), and oxygen (1.0 slm, Air Liquide Deutschland GmbH, Düsseldorf, Germany, 99.999%) were mixed and used during the 15 min deposition step, together with a substrate temperature of 923 K. A 15 min annealing step under the same gas atmosphere and temperature was added after synthesis. To reduce carbon surface contaminations that originate from the use of carbon-rich precursor materials, an additional annealing step was added. Thus, the synthesized films were subjected to 923 K for 1 h under ultrahigh vacuum conditions (~10^−7^ Pa) to remove these contaminations.

### 2.2. LiF Coating via Gas-Phase Fluorination

Surface fluorination of LLZTO films was performed by decomposing 2 g of polyvinylidene fluoride (PVDF) in a tube furnace under argon atmosphere with an argon gas flow of Q=1 slm and a temperature of 240 °C. This temperature was chosen in accordance with previous studies used for the fluorination of perovskite-based samples [CITATION]. Since the polymer is used in large excess compared to the amount of protective layer to be formed on the LLZTO surface, this reaction temperature must stay low to keep the degree of polymer decomposition low. The sample was transferred into the already heated tube furnace, kept at 240 °C for 5 min, and then taken out of the furnace directly into a glovebox with argon atmosphere (Air Liquide Deutschland GmbH, Düsseldorf, Germany, 99.999%) for cool down. The LLZTO thin films were placed in the gas stream behind the PVDF. By decomposing PVDF, small amounts of HF can reach the LLZTO surface, where the HF can then subsequently react with the surface contaminations like Li_2_CO_3_ or the also-detected LiOH.

$$\begin{array}{c} {\mathrm{Li}}_{2}{\mathrm{CO}}_{3}+2\;\mathrm{HF}\;\to 2\;\mathrm{LiF}+{\mathrm{CO}}_{2}+H_{2}O\\ \mathrm{LiOH}+\mathrm{HF}\;\to \mathrm{LiF}+H_{2}O \end{array}$$

The residuals are carried away by the argon gas stream. Two samples were prepared by coating LLZTO via gas-phase fluorination in order to check for influences of the fluorination time. The two time steps were done in 5 min and 15 min, respectively. Despite being prepared under UHV conditions and therefore being free of Li_2_CO_3_, the films still have some LiOH impurities on the surface, as the XPS investigations revealed. Furthermore, the transport to the tube furnace was done under argon, but during the insertion into the furnace the thin films were exposed to air, and thus small amounts of Li_2_CO_3_ and LiOH could form.

### 2.3. LiF Thin Films via Sputtering

LiF films prepared by sputtering were done using a RF-magnetron sputter process with a commercial LiF target (Alineason Materials Technology GmbH, Frankfurt am Main, Germany) at a pressure of $p=5\times 10^{-1}\;\mathrm{Pa}$ and an argon gas flow of Q=10 sccm.

### 2.4. PEO and LiTFSI Evaporation

For the deposition of thin films of poly(ethylene oxide) (PEO) and lithium bis(trifluoromethanesulfonyl)imide (LiTFSI) via physical vapour deposition, thermal co-evaporation from two independent sources was used. Each source consists of a self-made Knudsen cell with an electrically heated Al_2_O_3_ crucible. The PEO source is located directly under the garnet substrate, and the LiTFSI source is positioned at 45° to the PEO source at a distance of around 14 cm from the garnet film. PEO (2000 g/mol, Alfa Aesar, Karlsruhe, Germany) and LiTFSI (Sigma-Aldrich (Merck), Darmstadt, Germany) are used. Both PEO and LiTFSI are vacuum-dried at 10^−6^ Pa for at least 24 h prior to use. Evaporation is performed with an external temperature of the crucible at T_crucible_ = 483 K for PEO and T_crucible_ = 518 K for LiTFSI at a pressure between 10^−5^ and 10^−6^ Pa.

### 2.5. LLZTO Pellet Preparation

LLZTO powders with the nominal composition similar to that of thin films were prepared via the conventional solid state synthesis route. Stoichiometric amounts of Li_2_CO_3_ (Thermo Fisher, Karlsruhe, Germany >99.9%), La_2_O_3_ (Sigma-Aldrich, >99.9%), ZrO_2_ (Sigma-Aldrich (Merck), Darmstadt, Germany, >99%), and Ta_2_O_5_ (Alfa Aesar, Karlsruhe, Germany >99.9%) were weighed and hand-milled together with mortar and pestle. A 20 % excess of Li_2_CO_3_ was added to compensate for the lithium loss during sintering. Before use, La_2_O_3_ was dried at 1100 °C for 24 h to remove any moisture. The mixture was first calcined at 900 °C for 8 h at the heating and cooling rate of 3 °C/min. After this first heating cycle, the materials were ground again, and 20% of Li_2_CO_3_ was added again to compensate for lithium loss at high temperatures. The powders were then pressed into pellets by applying a pressure of 2 tons for a minute, followed by heat treatment at 1120 °C for 12 h with the heating and cooling rate of 3 °C/min under the ambient atmosphere. The obtained LLZTO pellets were then removed from the furnace at 500 °C and transferred to an Ar-filled glove box to minimize exposure to the environment.

### 2.6. X-ray Photoelectron Spectroscopy (XPS)

XPS measurements have been performed in a *PHI Versaprobe 5000* spectrometer by Ulvac-phi Inc., Kanagawa, Japan, with monochromatized Al Kα radiation (hν = 1486.6 eV), a spot size of d = 200 µm, and a power of P = 50 W. Pressure in the measurement chamber was around $p=10^{-7}\;\mathrm{Pa}$. Survey spectra were collected with a pass energy of $E_{pass}=187.85\;\mathrm{eV}$ and a step size of $E_{step}=1.0\;\mathrm{eV}$, while detailed spectra were collected with a pass energy of $E_{pass}=23.5\;\mathrm{eV}$ and a step size of $E_{step}=0.1\;\mathrm{eV}$. The angle between sample and analyzer was, if not stated otherwise, at $\theta =45^{{}^{\circ}}$. Interface experiments were performed by applying only slight amounts of material to the surface and then transferring the sample in situ to the XPS machine without breaking the vacuum for the subsequent measurements. This procedure was repeated until a covering film could be achieved and no substrate signal could be seen in the XPS anymore.

### 2.7. X-ray Diffraction (XRD)

XRD patterns were recorded with a Rigaku Smartlab device with a HyPix-3000 detector in Bragg-Brentano geometry with a Cu Kα source operated at 40 kV and 30 mA. For the LLZTO thin films, the scan was performed from 15° to 30° (the short range was used to minimize the measurement time in order to prevent degradation due to air leakages but is sufficiently large to identify impurity phases according to our experiences) with a step size of 0.005° and a fixed divergent slit with 0.3°. Due to the air sensitive nature of the measured thin films, airtight sample holders were used. For the LLZTO pellets the measurement rage of 10° to 80° was used, with a step size of 0.005° and a fixed divergent slip of 0.3°. Rietveld refinements were performed with TOPAS V6 (Bruker AXS, Karlsruhe, Germany) software.

### 2.8. Electrochemical Impedance Spectroscopy

Alternating current (AC) electrochemical impedance spectroscopy was carried out to characterize the conductivity of the as-synthesized and surface-treated LLZTO pellets. The pellets were sputtered with a thin layer of gold on both sides for electronic contacting. The impedance measurements were carried out in the temperature range of 30 °C to 100 °C, using the ITS unit (Biologic) connected to an MTZ-35 impedance analyser by applying an AC signal of 100 mV amplitude with the frequency ranging from 1 MHz to 100 mHz. Fitting of the data was performed using RelaxIS (rhd instruments, Darmstadt, Germany).

## 3. Results and Discussion

### 3.1. Surface Modification and Protection of LLZTO Thin Films via a Gas-Phase Fluorination Approach

The garnet films of nominal composition of Li_6.5_La_3_Zr_1.5_Ta_0.5_O_12_ could be obtained via laser-assisted chemical vapour deposition, similar to what was reported previously [19,20]. The advantage of this method for surface analysis originates from the fact that high purity garnet films with smooth surfaces suitable for subsequent surface-sensitive analysis methods can be obtained, which has shown to be suitable for various interface studies before [3,8]. The phase purity of the garnet film was confirmed via the Rietveld analysis of X-ray diffraction of data (see Figure S1). For the LLZTO thin film, the lattice parameter was 12.95 Å and the crystallite size was of the order of <100 nm. Apart from the garnet reflections, one could also identify the reflection belonging to silicate phases resulting from an oxidation of the Si substrate used to grow the LLZTO thin films, as observed previously [19]. Additionally, an exemplary micrograph of a sample coated under the same conditions, as the used one is shown in the SI to show the typical film thickness of the synthesized films, resulting in a load density of 0.28 mg/cm² (Figure S2).

**Figure 1:**
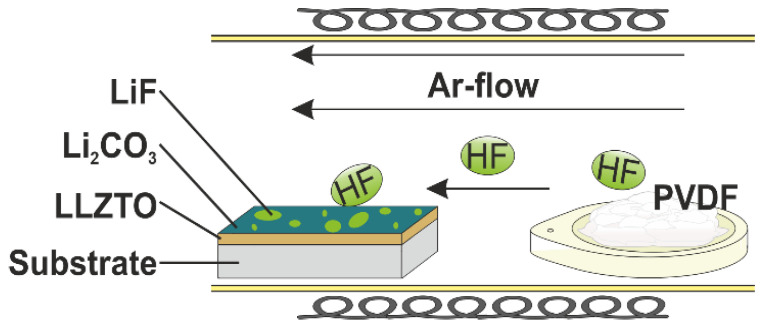
Schematic synthesis setup for the gas-phase fluorination.

To attempt a novel preparation route for surface fluorination under LiF formation, the films were placed inside a tube furnace, which was quickly heated to 240 °C, held at this temperature for 5 min, and quickly cooled afterwards, all under a flow of argon (99.999% purity). A PVDF-filled boat was placed in front of the LLZTO films (with respect to the flow direction), as shown in Figure 1.

**Figure 2:**
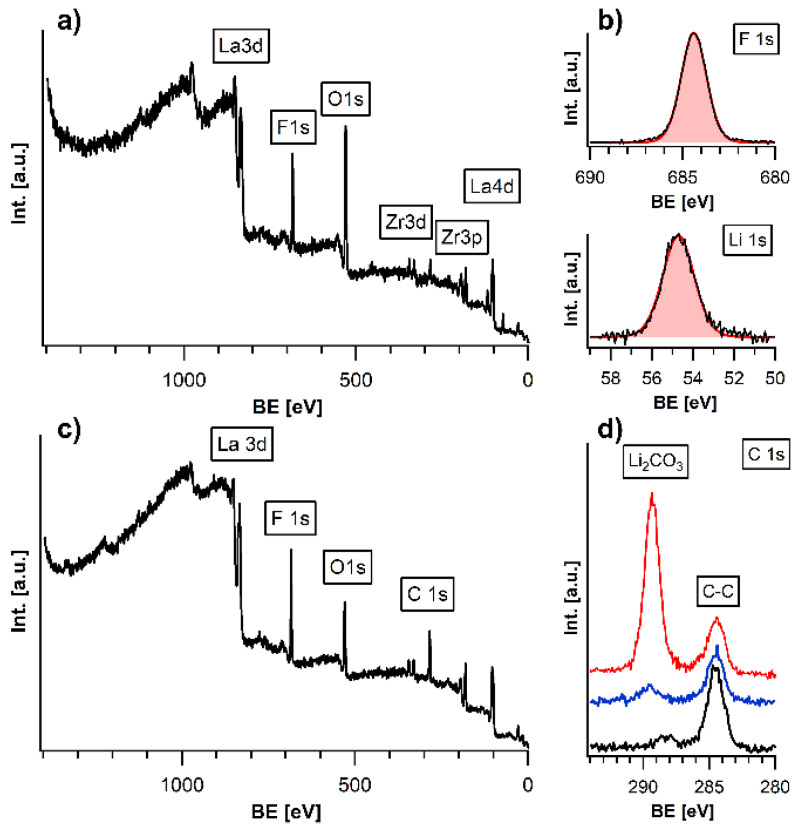
(**a**) XPS survey spectrum of gas-phase fluorination LiF coated LLZTO. (**b**) Detailed Li1s and F1s spectra of gas-phase fluorination LiF coated LLZTO. (**c**) XPS survey spectrum of gas-phase fluorination LiF coated LLZTO after 24 h in ambient air. (**d**) Comparison of the detailed XPS C1s emission lines of an uncoated LLZTO (red), sputter-coated LLZTO (blue), and gas-phase fluorinated LLZTO (black) after air exposure.

X-ray photoelectron spectroscopy was used to analyse the surface of the LLZTO films. Analysis of the pristine LLZTO film after deposition is shown in SI (Figure S3). A strong signal of F can be detected at the surface (Figure 2a). Remarkably, no carbon species apart from adventitious carbon (resulting from air exposure) could be found at the film surface, indicative of the HF-based fluorination mechanism (Figure S4). The formed LiF layer shows an approximate composition of Li_1_F_1.04_ in XPS measurements, confirming that fluoride mainly exists at the surface in a stoichiometric composition (Figure 2b)). Apparently, specific signals from the underlying garnet, such as the La3d and Zr3d peaks, as the most intense signals of LLZTO, are also still detectable. Additionally, the ratio of La:Zr was determined from XPS to be 66.6:33.4, still matching well with the composition of the garnet material. The overall intactness of the garnet film could also be confirmed by XRD analysis, confirming insignificant decomposition of the garnet as well as the absence of reflections belonging to LiF, for which the layer thickness is far too low to be detected by this method (Figure S1). Due to the surface sensitive character of the XPS measurement with an information depth below 10 nm, this implies that the formed layer of LiF must be very thin, in the range of a few atomic layers, or incompletely covering the surface. We further note that the exposure time to PVDF does seem to have a strong influence on the thickness of the LiF layer. This is indicated from the observation that after tripling the exposure time, the La/Zr/Ta LLZTO signals cannot be detected by XPS anymore (Figures S4 and S5), which implies that the thickness of the LiF film has increased to >10 nm and shields the elements from below due to the surface sensitivity of XPS. This also implies that the reaction time has to be chosen carefully in the order of 15 min to limit the thickness of the LiF layer formed.

In order to compare the gas-phase fluorination approach to other surface modification techniques, we use a sputtered LiF film as comparison. Thus, we compared gas-fluorinated films to LiF-sputtered films with respect to their surface composition, as well as their behaviour on air exposure. Additionally, for the LiF sputtered films, the resulting Li:F ratio of 50:50 confirms the presence of LiF at the surface, while La and Zr from the underlying substrate are still detectable (Figure S6). Comparing the two methods, the gas-phase fluorination is advantageous regarding the required reaction time (5 min vs. 120 min), as well as the fact that no high vacuum equipment is needed.

In order to examine the protective character of the LiF and to determine the degree of coverage, the different LiF-coated LLZTO films were exposed to ambient air for 24 h and compared to an un-coated film. For the uncoated, a strong signal corresponding to Li_2_CO_3_ at 289.55 eV [21] can be found in the C1s spectrum (next to the signal of adventitious carbon, which originates from the need to remove the film from UHV conditions, see red curve in Figure 2d), as is expected due to the strong basicity of the material on exposure to CO_2_/H_2_O. Furthermore, no signals of the heavy elements can be detected at the surface, showing ongoing degradation due to the absence of surface passivation (see Figure 2d and Figure S7). In contrast, the gas-phase fluorinated film (see red curve in Figure 2d) shows no formation of Li_2_CO_3_ without indication of degradation of the LiF-layer and the LLZTO layer underneath after the same exposure to air. The observation that the garnet phase below the LiF film can be detected by XPS also indicates why LiF itself is hard to be detected using diffraction methods. Due to its low thickness and low scattering strength for the LiF-coated films, all characteristic peaks of the LLZTO are still detectable in the XPS survey spectrum (Figure 2c), indicating that the garnet phase corresponding to these elements is still intact. On comparing the detail spectrum of the C1s emission line, the gas-phase fluorinated sample did not show signs of a carbonate signal, whereas the surface-sputtered film showed a small signal belonging to Li_2_CO_3_ (Figure 2d). The increased surface stability to ambient conditions can be well explained by the low basicity of LiF, which has a high formation energy [22] and does not form hydrate phases [23] as compared to highly basic LLZTO, which reacts with acidic compounds such as H_2_O/CO_2_. Further, it can be concluded that the gas-phase fluorinated films possess a close to full surface coverage, whereas the sputtering can easily lead to partially incomplete covering due to shadowing effects, possibly induced by a small roughness of the synthesized LLZTO films.

In addition, attempts were made to study the influence of the LiF coating on the Li-ion conductivity by electrochemical impedance spectroscopy. Applying this technique to thin films is an experimental challenge. We emphasize that it was not possible to conduct this study with the LLZTO thin films due to the use of non-conducting SiO_2_ substrates, which would only allow for in-plane measurements with an unfavourable surface to volume ratio to determine the influence of the LiF coating. Further, the use of alternative conducting substrates such as Pt results in the formation of Li_2_PtO_3_ at the Pt to garnet interface, which possess an impedance signature [19] and would overlap with additional surface layer impedances from surface fluorination.

**Figure 3:**
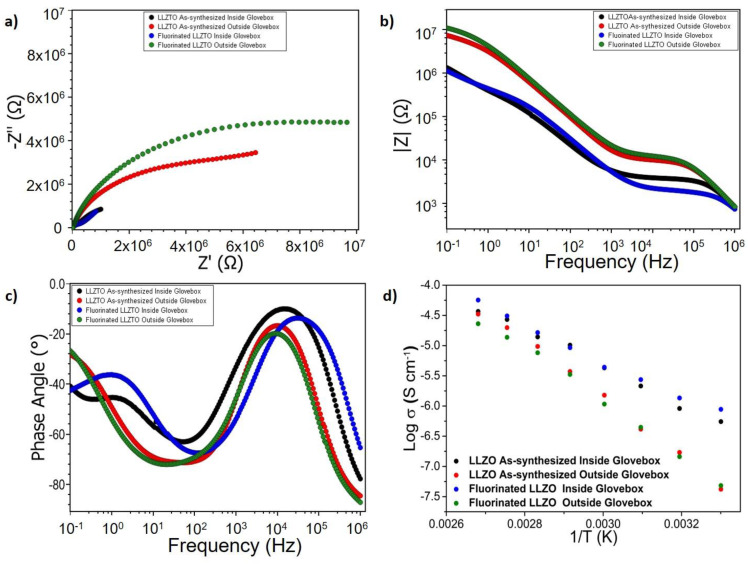
Comparison of the (**a**) Nyquist. (**b**,**c**) Bode plots of the as-synthesized and fluorinated LLZTO pellets kept inside and outside of the argon filled glovebox. (**d**) Arrhenius plots for the same samples measured in the temperature range of 30 °C and 100 °C.

To avoid these effects and to facilitate evaluation of the impedance data, we applied the developed fluorination method to a series of sintered garnet pellets with the theoretical densities of ~74%. By this, we attempted to investigate pellets of the pure garnet material to pellets which were fluorinated with the PVDF-based gas-phase route on both sides, but not exposed to air. In addition, a second set of both fluorinated and non-fluorinated pellets were prepared and left standing outside the glovebox for ~1 day to induce humidity and CO_2_-related degradation in order to develop some understanding on the morphology of the fluorinated surface and its application potential. From the recorded powder X-ray diffraction data (Figure S8) and the corresponding Rietveld fits, the lattice parameters of all the composition studied were found comparable to each other and to the LLZTO thin film. The lattice parameters for the LLZTO pellets are given in Table S1. A comparison of the recorded impedance data is shown in Figure 3 (exemplary fits of the impedance data are shown in Figure S9, the corresponding equivalent circuit models are shown in Figure S10).

XRD analysis (which characterizes the first 100 µm of the pellet surface at maximum) shows that the fluorination is not impacting the bulk structure of the garnet materials (see Figure S8). From the Nyquist and Bode plots in Figure 3a–c, as well as the evaluation of the temperature dependency (see Figure 3d), one can derive some information on the impact of the fluorination reaction on functional properties. The impedance spectra of fluorinated and non-fluorinated pellets, which have not been left standing outside, are basically identical (black and blue curve in Figure 3c,d). Remarkably, there is no indication of a clear separate semicircle appearing for the fluorinated comparing to the non-fluorinated sample, which indicates that only minor transport resistances would be induced when keeping the fluorination time low. Thus, we conclude that the fluorination reaction does not result in a degradation of the underlying garnet material and that the LiF is likely to be present at the surface only. This surface layer can be expected to be thin as obtained for the thin films and not impede the conductivity significantly more than surface phases originating from small off-stoichiometries at the surface obtained after garnet synthesis would do anyways. This can be brought into good agreement with the XPS spectra discussed in the previous section, which shows that the surface layer formation of LiF via a gas-phase approach requires a well-adjusted reaction time but can then lead to transformation of surface species to LiF without destruction of the underlying garnet phase.

Further, the resistance of both fluorinated and non-fluorinated pellets increases significantly once they are left standing outside. At first, this appears to be counterintuitive to the XPS spectra of the thin films reported shown in Figure 2d, which do not indicate carbonate formation. However, the porous nature of the pellet must be taken into account. Thus, the impedance study shows that though the LiF surface layer is stable under ambient condition [5], it cannot protect degradation of the garnet from gas-phase degradation fully if the substrate shows high porosity. This can be understood regarding the fairly low density of the pellet of ~74% of the theoretical density (see Figure S8 for the XRD patterns of the pellets). This indicates that although the method is suitable for modifying surfaces, the streaming profile within the tube furnace might be unfavourable for protecting inner pores fully from interaction with H_2_O/CO_2_. Thus, applying this method to modify porous LLZTO pellets before infiltration with PEO+LiTFSI needs to be elaborated further [24].

### 3.2. Analysis of the Stability of the LLZTO and PEO/LiTFSI Interfaces towards LiF

Due to the potential of LLZTO to be used in combination with polymer electrolytes such as PEO/LITFSI, we further characterized the potential existence of reaction layers at the interface between LiF and LLZTO as well as PEO/LiTFSI. Such reaction layers can have an important impact on functional properties in case of formation of unfavourable side phases which might hinder lithium-ion migration through the interface by passivating phases. Due to the fact that the gas-phase fluorination route cannot control the thickness of the LiF layer, these experiments had to be performed on sputter-coated thin films, which allow for precise adjustment of LiF layer thickness. Prior to discussing the results, we would like to make the reader aware of the following aspect: LiF is, in principle, a very stable compound, with a high formation energy and stability towards the formation of hydrate phases [22]. However, the study presented in the following addresses the surface of LiF particles and not a bulk degradation of LiF; the surface has a higher energy than the bulk and can thus perform reactions in order to minimize the surface energy of LiF, and these reactions are hard to predict.

**Figure 4:**
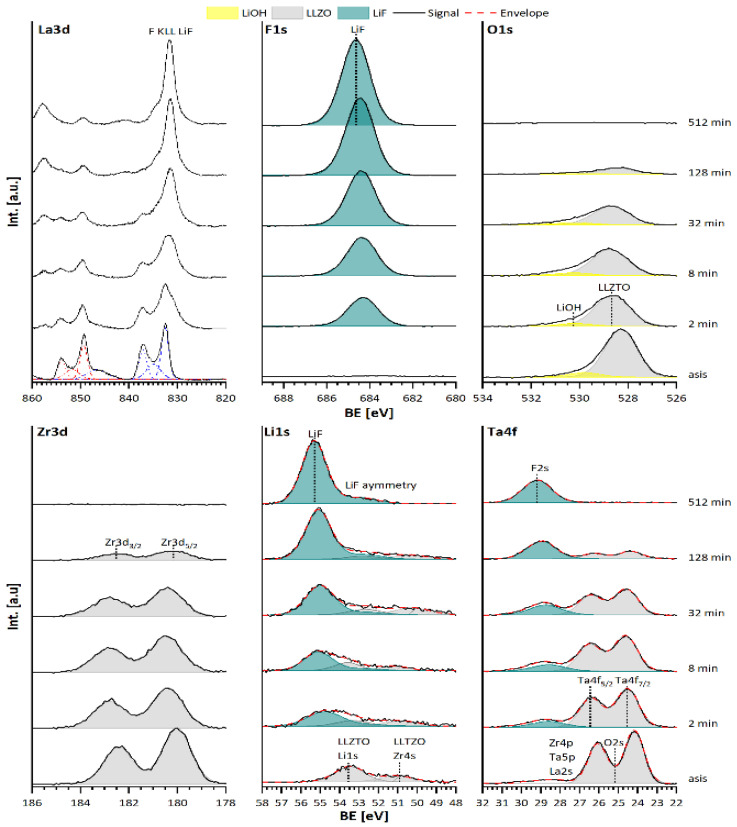
La3d, F1s, O1s, Zr3d, and Ta4f XP spectra of the interface between LLZTO and LiF, where “asis“ denotes the annealed LLZTO substrate deposition times of LiF from 2 min up to 512 min.

The XP spectra of the annealed (asis) LLZTO substrate show a typical LLZTO emission line (Figure 4, with similar line shape and position to what has been previously for LLZTO by our group [8]). After the annealing process the surface of the LLZTO is completely free of any carbon contamination (Figure S11), and only a small amount of LiOH is indicated (see Figure 4 O1s emission). None of the La3d, Zr3d, or Ta4f emission lines show a shift of position, which would indicate reduction or oxidation from the sputtering process. After sputter deposition of LiF with 2 min, the F1s spectrum shows only the emission line of LiF at its typical binding energy of 684.7 eV [25,26]. While the shape of all LLZTO emissions does not change upon LiF deposition, an overlap between the La3d emission line and the fluorine KLL Auger line of the LiF between the binding energies from 825 and 840 eV [27] is indicated. In the O1s and the Zr3d spectra no new emission lines can be observed, with additional damping of the LLZTO and LiOH intensities indicating increasing coverage of the film surface. The Li1s spectrum now exhibits the Li1s emission line of LiF at 55.0 eV. The Ta4f lines are damped as well, and the appearance of the F2s line of LiF at 28.9 eV can be observed. Upon increasing the LiF deposition time to 32 min, the same trend as for after 2 min can be observed, and proceeds further when increasing the deposition time to 512 min. For this time, the spectra do not show any LLZTO emission lines, and only the emission lines of the lithium (Li1s) and fluorine (F1s, F2s, F KLL) are present. The missing O1s emission line in this spectrum demonstrates the complete absence of any oxygen as well. During the deposition, both the LLZTO and the LiF emission lines undergo a binding energy shift (Figure 4). This can be attributed to intercalation of lithium-ions into the LLZTO at the beginning of the LiF deposition. A detailed discussion of this phenomenon can be found in Section 3.2.1. Overall, the interface is not reactive in any form, nor does it show the formation of permanent space charge layers.

**Figure 5:**
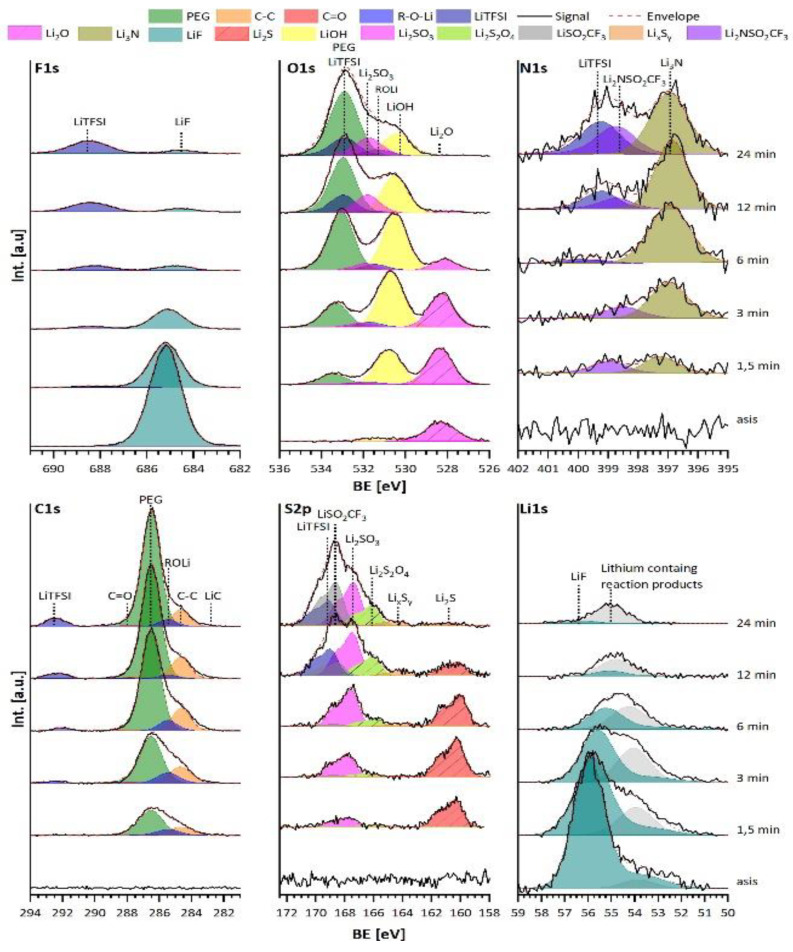
F1s, O1s, N1s, C1s, S2p, and Li1s XP spectra of the interface between LiF and PEG+LiTFSI, with deposition times from 1.5 min up to 24 min.

The second interface investigated is between a LiF film, with PEO+LiTSFI as a solid polymer electrolyte (SPE). This is performed with the thermal evaporation process of the oligomer PEG with a molecular weight of 2000 g/mol and the conductive lithium salt LiTFSI as introduced by Ferber et al. [28]. Figure 5 shows the “asis” denoted spectra originating from the freshly synthesized LiF layer including the PEO+LiTFSI deposition with deposition times between 1.5 up to 24 min. The bare LiF layer shows its typical emission lines with the F1s at 685.2 eV and Li1s at 56.0 eV. Additionally, a small amount of Li_2_O is present, as is evident from the emission line in the O1s spectrum at 528.3 eV [29,30]. No emission lines can be found in the N1s, C1s, or the S2p spectra.

With the start of the PEO+LiTFSI deposition at 1.5 min, the F1s emission line of LiF is damped in its intensity, while a new smaller emission line at a binding energy of 688.1 eV appears, which can be attributed to the CF_3_ group of the LiTFSI [31]. The O1s spectrum now consists of three clearly distinguishable emission lines from the PEO and LiTFSI at 533.4 eV [31,32,33], LiOH at 531.3 eV [30,34], and the already present Li_2_O. In addition, the emission lines for the R-O-Li and Li_2_SO_3_ at 531.8 and 532.2 eV can be found, which show corresponding signals in the C1s and S2p spectra [29,35]. In the N1s spectrum, two emission lines are indicated to originate from Li_3_N and Li_2_NSO_2_CF_3_ (as later shown) at 397.3 and 399.0 eV, respectively [26,30,36]; the C1s spectrum shows a main emission line from PEO at 286.5 eV, accompanied by R-O-Li and hydro carbons at 285.4 and 284.7 eV [32,33]. Lastly, the S2p spectrum only exhibits the two emission lines from Li_2_S and Li_2_SO_3_ at 160.3 and 167.8 eV [31,35].

At this point it is clear that the LiTFSI is significantly decomposed and has reacted at the surface of the LiF, since no LiTFSI emission lines could be detected. The authors think that this likely results from a surface stabilization of the LiF surface. In contrast, the typical emission lines for the PEO can be detected, which leads to the assumption that it remains mostly intact. However, we acknowledge that it is not straightforward to deduce smaller degrees of reaction for the PEO, since the carbon-containing products may originate from LiTFSI only or LiTFSI and PEO together.

After 6 min of deposition, a similar behaviour was observed. For F1s spectrum the LiF and LiTFSI emission lines are now equally intense, the O1s spectrum is dominated by the PEO and LiOH emission lines, the N1s consists mostly of Li_3_N and minor amounts of LiTFSI, the C1s shows one strong emission for PEO accompanied by R-O-Li and hydrocarbons and a smaller LiTFSI emission, while the S2p mostly shows Li_2_SO_3_ and Li_2_S, but now also emission lines for Li_x_S_y_ and Li_2_S_2_O_4_ at ~163–164 eV and 165.9 eV, respectively [35,37,38]. After 24 min there is mainly the LiTFSI emission line with a small amount of LiF in the F1s spectrum, which is attributed to the X-ray beam damage of the LiTFSI in PEO and might not originate from the LiF substrate itself [28,39]. The O1s spectrum is deconvoluted on the basis of the N1s, C1s, and S2p spectra, consisting mainly of PEO, LiTFSI, Li_2_SO_3_, and LiOH emissions. For the N1s spectrum, three emissions lines from Li_3_N, Li_2_NSO_2_CF_3_, and LiTFSI are visible. Further, the C1s spectrum shows an increased amount of PEO, LiTFSI, and hydrocarbons, and minor amounts of LiC around binding energies of ~283 eV [26]. Finally, the S2p spectrum now shows the typical emission lines for LiTFSI and LiSO_2_CF_3_ at 169.0 and 168.7 eV alongside the already present compounds [40]. Only the amount of Li_2_S is drastically reduced. The fact that the degradation of LiTFSI is impeded for longer deposition times indicates that the interface between LiF and LiTFSI has stabilized, and that further reaction is impeded.

There are strong similarities not only in the kind but also in the order of the reaction products that are found at the interface between LiF and PEO+LiTFSI and the reduction of LiTFSI on lithium, as shown by Aurbach and Yildirim [41,42,43]. The weakest bond in the LiTFSI molecule is the S-N bond, and when cleaved by lithium, the two segments LiSO_2_CF_3_ and Li_2_NSO_2_CF_3_ form, which is indicated in the N1s and S2p spectra after 24 min. Additionally, the signals, which would match phases such as Li_3_N, Li_2_S, Li_2_SO_3_, and Li_2_SO_4_, can only originate from the decomposition of LiTFSI. We acknowledge that although the reaction products and their order hints towards a reduction reaction, a direct electron transfer can be fully excluded due to the large band gap of LiF of 13.6 eV [44], with the valence band maximum around 5.9 eV. This would result in a high valence band, with HOMO offset around of around 2 eV.

We propose a possible pathway for the reaction where the LiF forms HF from the remaining water in UHV, which may be present as indicated by the formation of LiOH out of Li_2_O. Alternatively, a further reaction of the HF with LiTFSI could lead to its decomposition.

The structure of the LiF layer may potentially have a strong influence on its reactivity, since reports of Li et al. [45] indicate that LiF, which was sputter deposited at room temperature, is amorphous and not crystalline. In addition, as already discussed, unsaturated bonds at the surface might lead to the higher reactivity of the LiF, resulting in a surface stabilization and explaining the strong surface limitation of the reaction.

We emphasize that a reaction which is driven by the thermodynamics of the surface cannot be derived upon standard formation enthalpy of all the reaction products and would require intensive theoretical modelling, which is out of the scope of this article.

Regardless of the driving force, it appears that the reaction is limited to a depth of few nanometres, since the binary lithium salts are only found at the very interface, and LiTFSI remains intact for ongoing deposition. Even though this reaction is unexpected, its impact on the functional properties might be low since the LiF layer can prevent further degradation. However, the possibility of the formation of interfacial space charge regions cannot be neglected and will require further investigations.

#### 3.2.1. Discussion of the Binding Energy Shift at the LLZTO-LiF Interface

**Figure 6:**
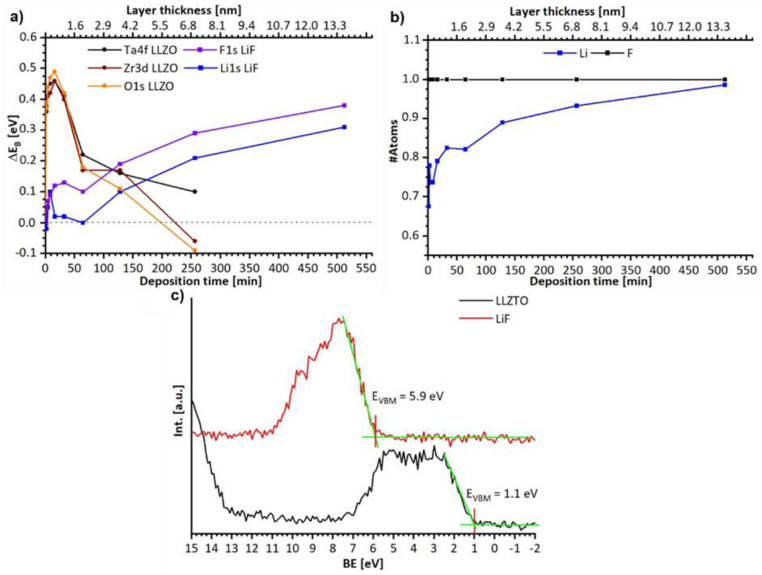
(**a**) Binding energy shift observed during the LLZTO-LiF interface experiment for LLZO and LiF on the basis of the Ta4f, Zr3d, and O1s positions for the LLZTO and F1s and Li1s for the LiF. (**b**) Lithium to fluorine ration determined by XPS during the interface experimented, referencing one fluorine. (**c**) Valence band spectra and EVBM position for LLZTO from the asis state and LiF after t = 512 min.

During the deposition process both, the LLZTO and the LiF emission lines undergo a binding energy shift, as shown in Figure 6. While the LLZTO signals shift to higher binding energies by ΔE_B_ = +0.5 eV after few minutes of deposition, they shift back to their original binding energy after 256 min. LiF shows a binding energy shift to higher binding energies up to ΔE_B_ = +0.3 eV after 512 min. After determining the layer thickness of the LiF on the basis of the Lambert–Beer law and the damping of the LLZTO O1s, Zr3d_5/2_, and Ta4f_7/2_ emission line, it becomes obvious that the positive binding energy shift of the LLZTO only occurs for LiF layer thicknesses below 1 nm. This behaviour might be explained by the intercalation of lithium-ions into the LLZTO (e.g., via a local space charge formation) due to the incomplete forming process of LiF. With ongoing growth of the LiF layer, the chemical potential of the lithium-ion is then reduced in the stabilized bulk LiF, and the modification of LLZTO might be reversed. Hence, a negative binding energy shift occurs in the LLZTO and a positive one in the LiF. This is, in principle, an agreement with the observed Li:F ratio shown in Figure 6b, which indicates an excess of fluorine in the beginning and stoichiometric LiF after 512 min. Again, any electron transfers can also be excluded by the large valence band offset of ΔE_VBM_ = 4.8 eV, as measured in Figure 6c, and the band gap of 13.6 eV for the LiF [44].

## 4. Conclusions

In this work, we show that gas phase fluorination can be a viable route for the formation of a protective and well-covering surface layer on top of suitable morphologies of garnet solid electrolytes and can be favourable compared to sputter-coating, which requires UHV conditions. Applying this method to flat garnet films, we found that the surface can be well protected from degradation in ambient air under the formation of Li_2_CO_3_. However, this method must be elaborated further to be applied to powder samples or porous pellets to understand diffusion limitations within the gas-phase, which would require optimization of heating time and temperature of substrate and/or PVDF precursor as well as trying different fluorine-containing polymers such as PTFE. In addition, insights on the behaviour of such LiF interlayers in composite electrolytes were elaborated, providing a detailed study of the interface between LLZTO, sputter-deposited LiF, and LiF with deposited PEO+LiTFSI. By this, we show that LiF appears to be fully stable towards LLZTO, but it can cause side reactions to PEO+LiTFSI, which is relevant for the understanding needed to improve and fabricate functional composite electrolyte materials with sufficient elastic properties.

## Data Availability

Data are available from the authors upon request.

## Supplementary Materials

The following supporting information can be downloaded at: https://www.mdpi.com/article/10.3390/ma15196900/s1, Figure S1. XRD of the LLZTO sample coated for 5 min with LiF via gas-phase fluorination. Figure S2. Exemplary SEM micrograph of a LLZTO film prepared under the same conditions as the used one to demonstrate the film thickness. Figure S3. Survey and detail spectra of the LLZTO thin film after synthesis and before gas-phase fluorination. Figure S4. (a) Detail spectra of the 5 min gas-fluorinated LLZTO after the fluorination step (b) Detail spectra of the 15 min gas-fluorinated LLZTO after the fluorination step (c) Detail spectra of the 5 min gas-fluorinated LLZTO after 24 h in air. Figure S5. XPS survey spectrum of the LLZTO film coated for 15 min with LiF via gas-phase fluorination. Figure S6. XPS survey of the LLZTO coated with LiF via sputtering for 120 min, note that the doublets are only labelled with the corresponding name of the doublet emission line. Figure S7. XPS survey of the uncoated LLZTO sample after 24 h in air. Figure S8. (Left) Diffraction analysis of the pellet surface of LLZTO pellets before and after fluorination, as well as after exposure to ambient conditions. (Right) Rietveld fit for the pristine LLZTO pellet. Small amount for LiAlO_2_ formation is due to the reaction of the pellet with the Al_2_O_3_ crucible. Figure S9. Fits of the Nyquist plot recorded at 30 °C (a,b) As-synthesized and fluorinated LLZO kept inside of the glove box, (c,d) As-synthesized and fluorinated LLZO kept outside of the glove box. Figure S10. Equivalent circuit models used for the evaluation of impedance data. (a) was used for the modelling the impedance data for the as-synthesized LLZTO pellet inside the glove box and also for both the as-synthesized and fluorinated LLZO pellet outside of the glovebox. (b) was used for fitting the impedance data of the fluorinated LLZTO pellets kept inside of the glove box. Figure S11. C1s detailed spectrum of the annealed LLZTO surface prior to the LiF interface experi-58 ment.
