# Supplementary material for: On the Surface Modification of LLZTO with LiF via a Gas-Phase Approach and the Characterization of the Interfaces of LiF with LLZTO as Well as PEO+LiTFSI
Source: Materials (Basel). 2022 Oct 5;15(19):6900. doi: 10.3390/ma15196900 (PMC9570571; doi:10.3390/ma15196900)
Supplement: Supplementary file 1 [file materials-15-06900-s001.zip › materials-1861697-supplementary.pdf]

materials

MDPI

Supporting Information

On the Surface Modification of LLZTO with LiF via a Gas-Phase Approach and the Characterization of the Interfaces of LiF with LLZTO as Well as PEO+LiTFSI

Manuel Donzelli [1,2], Thimo Ferber [3], Vanita Vanita [2], Aamir Iqbal Waidha [2], Philipp Müller [3], Maximilian Mellin [3], René Hausbrand [3], Wolfram Jaegermann [3] and Oliver Clemens [2,*]

1 Fachgebiet Materialdesign Durch Synthese, Fachbereich Materialwissenschaft, Technische Universität Darmstadt, Alarich-Weiss-Straße 2, 64287 Darmstadt, Germany
2 Chemische Materialsynthese, Institut für Materialwissenschaft, Universität Stuttgart, Heisenbergstraße 3, 70569 Stuttgart, Germany
3 Fachgebiet Oberflächenforschung, Fachbereich Materialwissenschaft, Technische Universität Darmstadt, Otto-Berndt-Straße 3, 64287 Darmstadt, Germany
* Correspondence: oliver.clemens@imw.uni-stuttgart.de; Fax: +49-711-685-51933

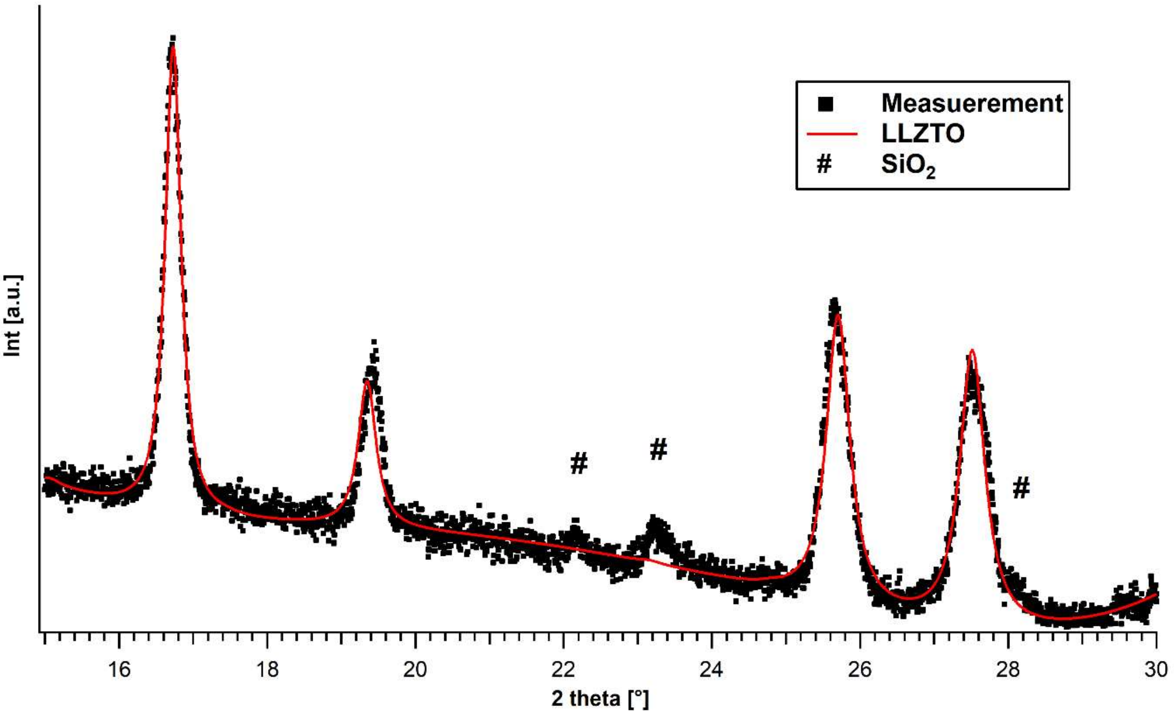

Figure S1. XRD of the LLZTO sample coated for 5 min with LiF via gas-phase fluorination.

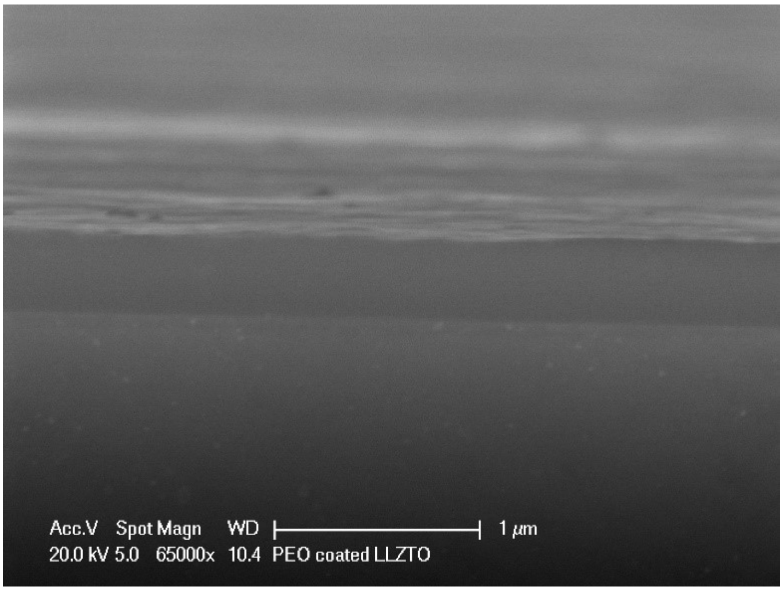

Figure S2. Exemplary SEM micrograph of a LLZTO film prepared under the same conditions as the used one to demonstrate the film thickness.

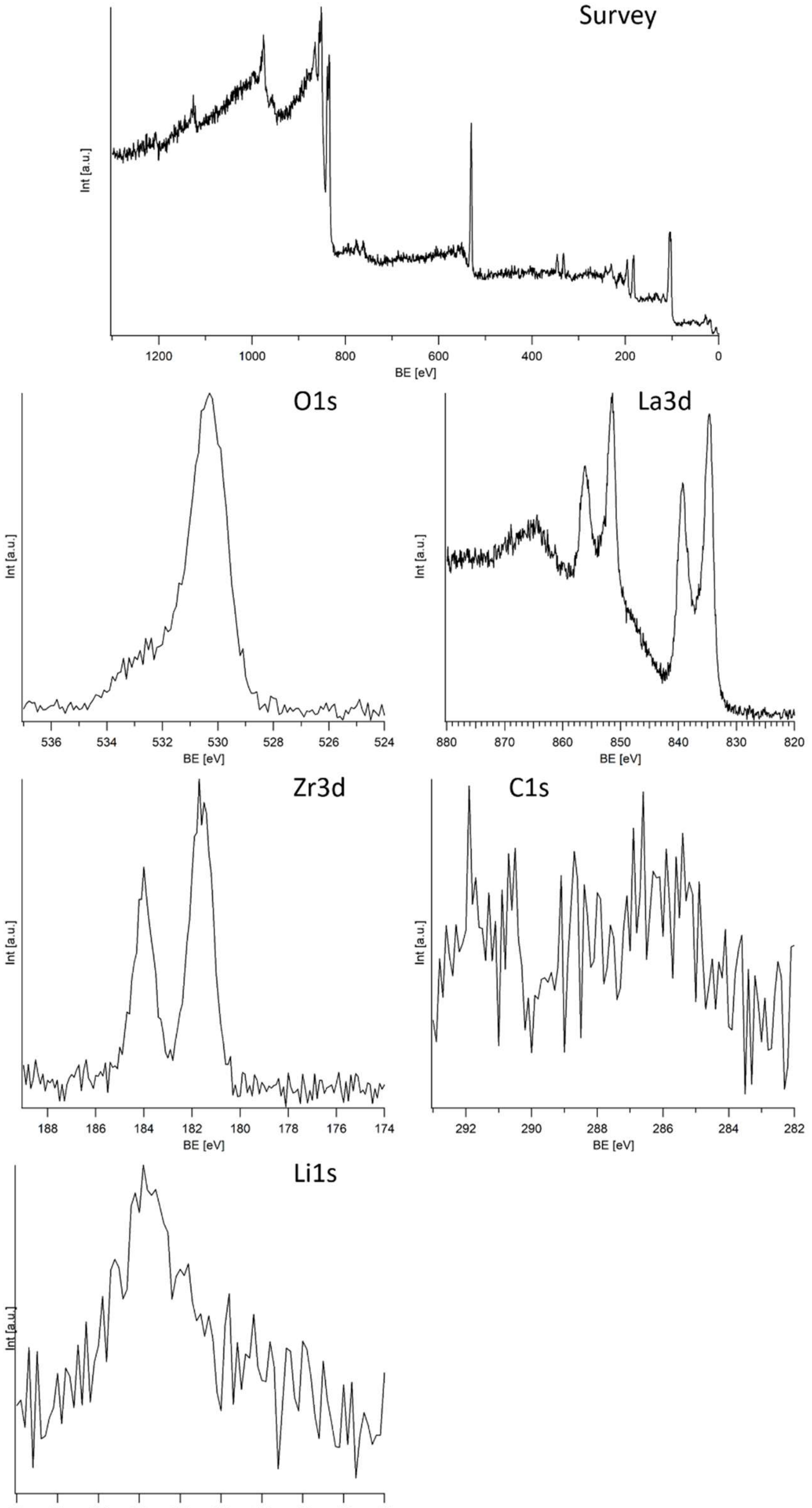

Figure S3. Survey and detail spectra of the LLZTO thin film after synthesis and before gas-phase fluorination.

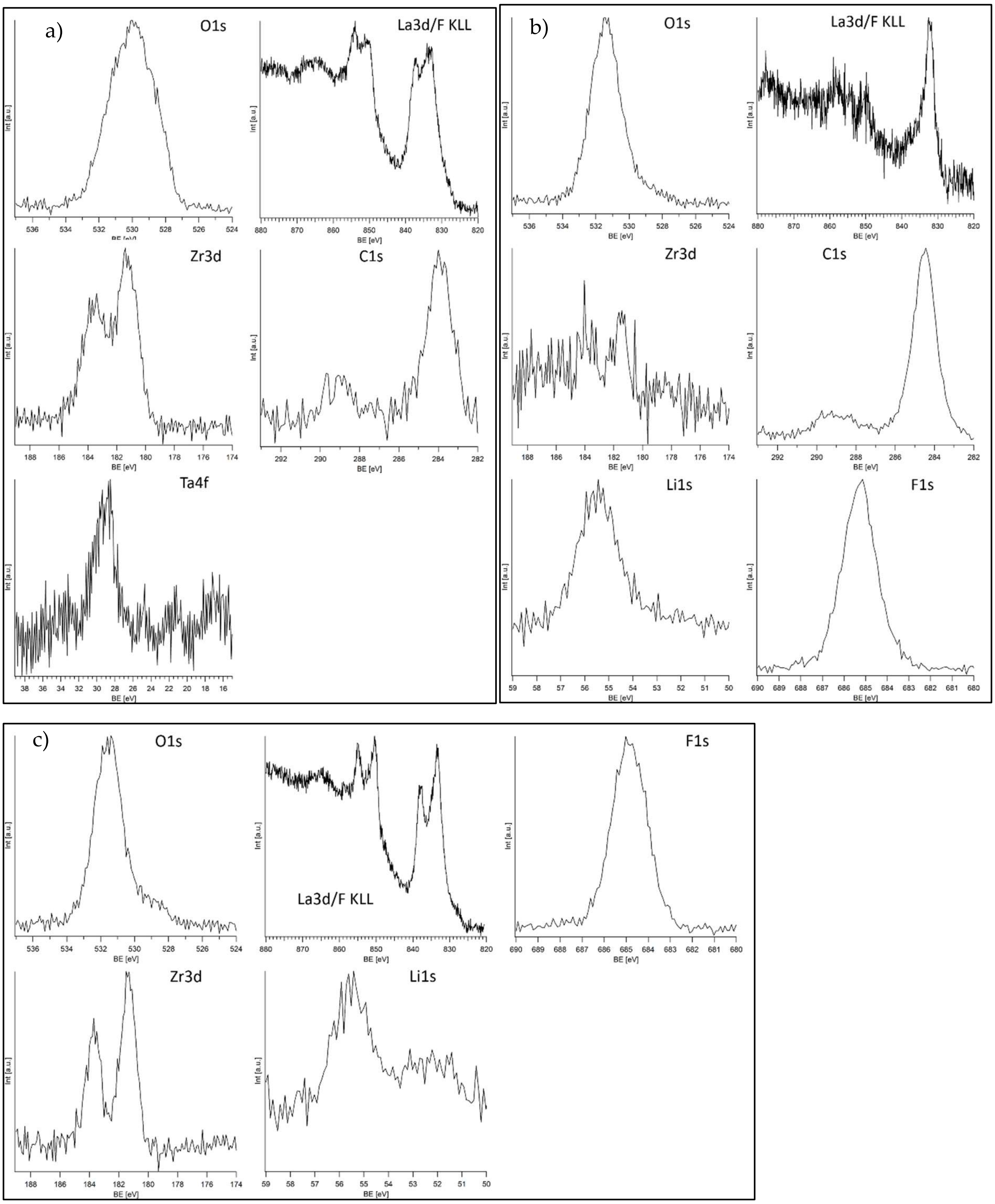

Figure S4. (**a**) Detail spectra of the 5 min gas-fluorinated LLZTO after the fluorination step (**b**) Detail spectra of the 15 min gas-fluorinated LLZTO after the fluorination step (**c**) Detail spectra of the 5 min gas-fluorinated LLZTO after 24 h in air.

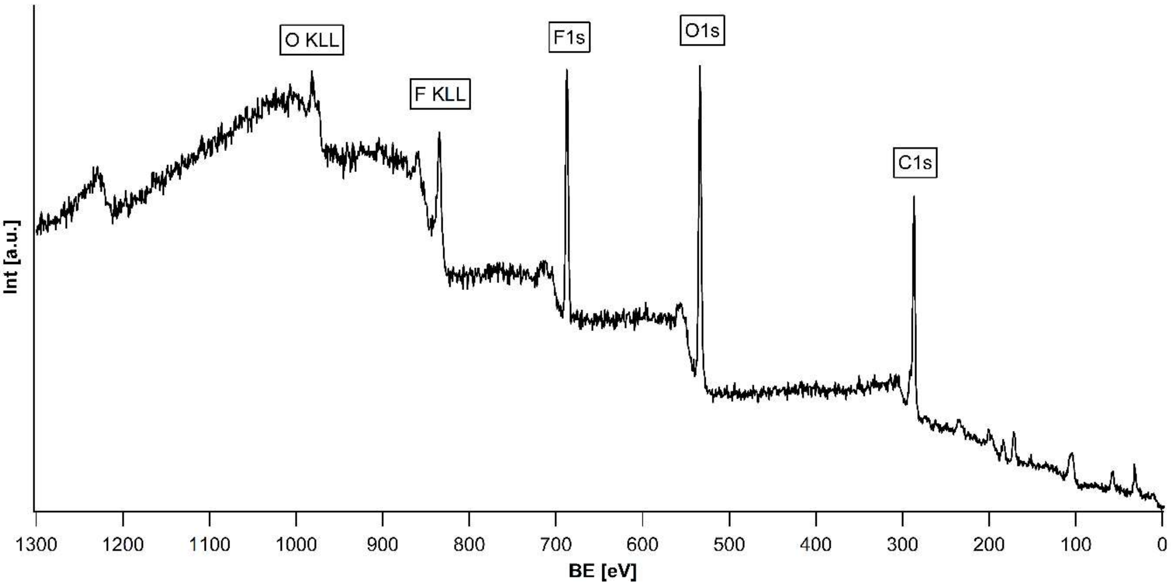

Figure S5. XPS survey of the uncoated LLZTO sample after 24 h in air.

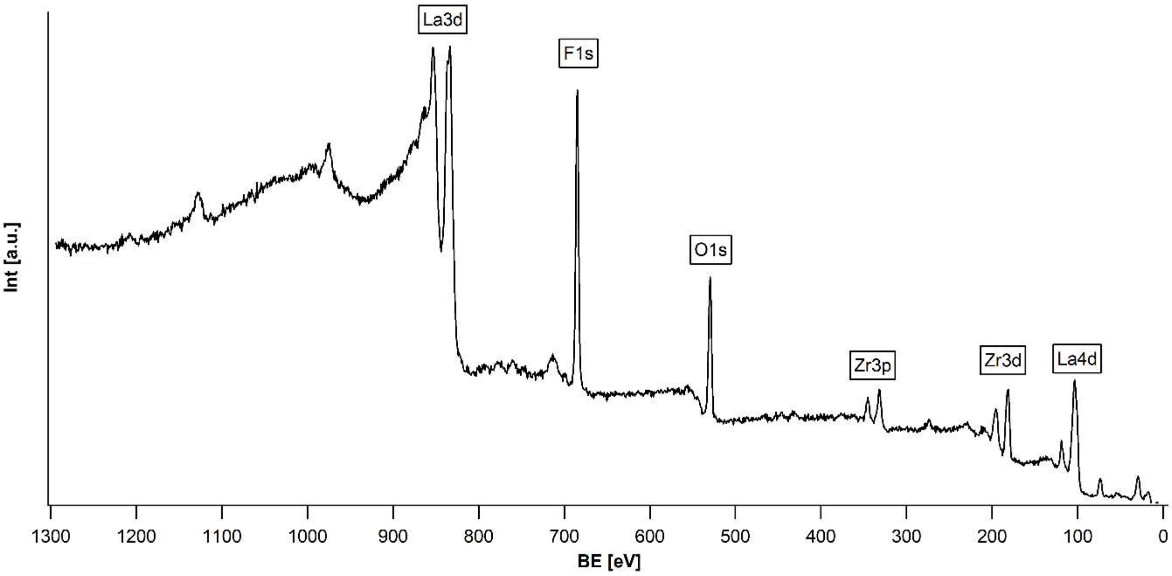

Figure S6. XPS survey of the LLZTO coated with LiF via sputtering for 120 min, note that the doublets are only labelled with the corresponding name of the doublet emission line.

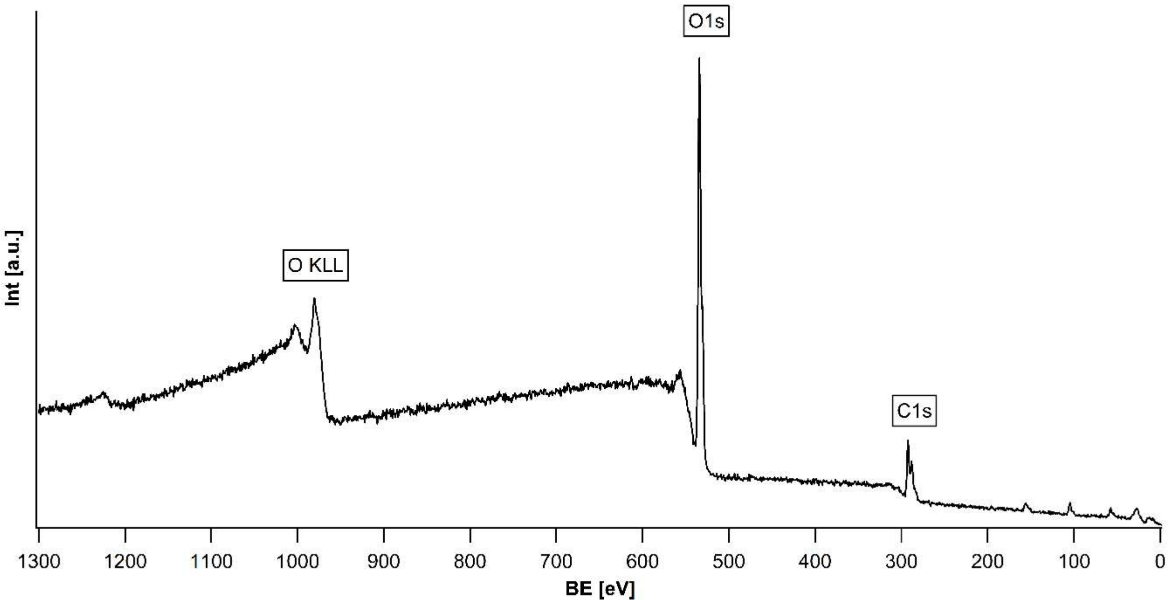

Figure S7. XPS survey of the uncoated LLZTO sample after 24 h in air.

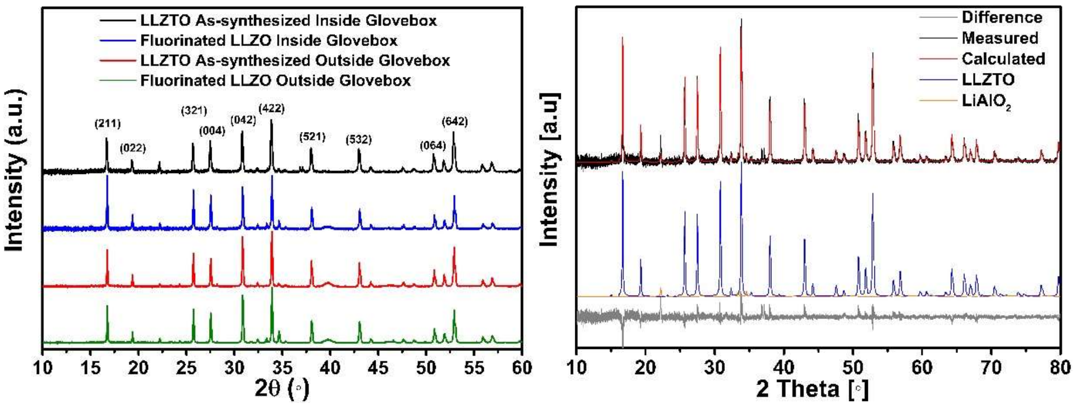

Figure S8. (Left) Diffraction analysis of the pellet surface of LLZTO pellets before and after fluorination, as well as after exposure to ambient conditions. (Right) Rietveld fit for the pristine LLZTO pellet. Small amount for $LiAlO_2$ formation is due to the reaction of the pellet with the Al_2O_3 crucible.

Table S1. Lattice parameters obtained for LLZTO pellets obtained from Rietveld refinements.

Pristine LLZTO	Fluorinated inside	Pristine LLZO Outside	Fluorinated Outside
12.93883(14)	12.93119(12)	12.93809(12)	12.93633(12)

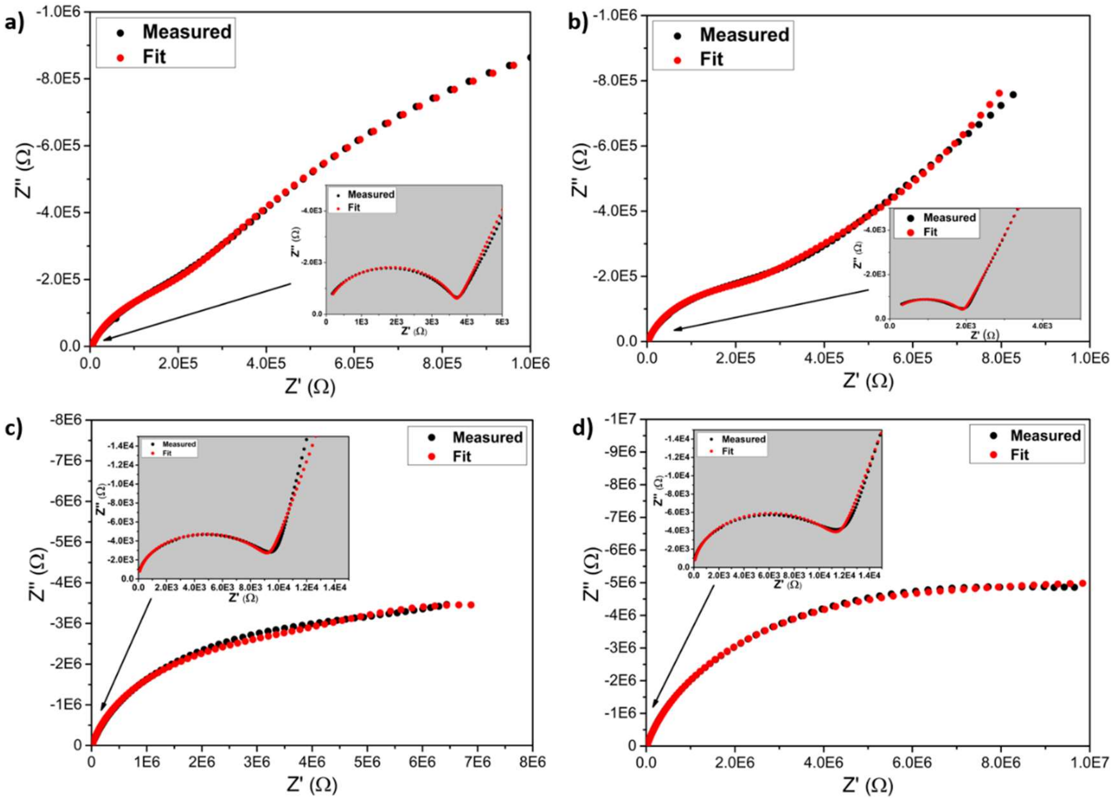

Figure S9. Fits of the Nyquist plot recorded at 30 °C (a,b) As-synthesized and fluorinated LLZO kept inside of the glove box, (c,d) As-synthesized and fluorinated LLZO kept outside of the glove box.

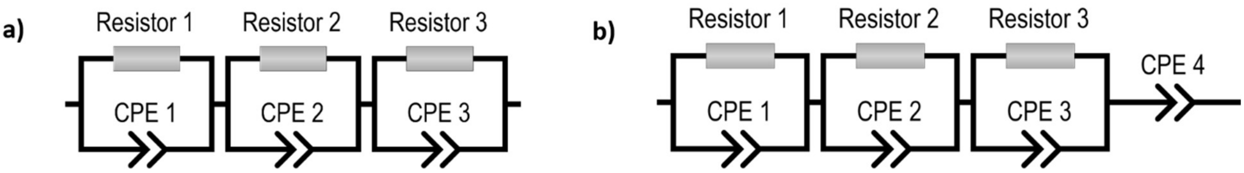

Figure S10. Equivalent circuit models used for the evaluation of impedance data. (a) was used for the modelling the impedance data for the as-synthesized LLZTO pellet inside the glove box and also for both the as-synthesized and fluorinated LLZO pellet outside of the glovebox. (b) was used for fitting the impedance data of the fluorinated LLZTO pellets kept inside of the glove box.

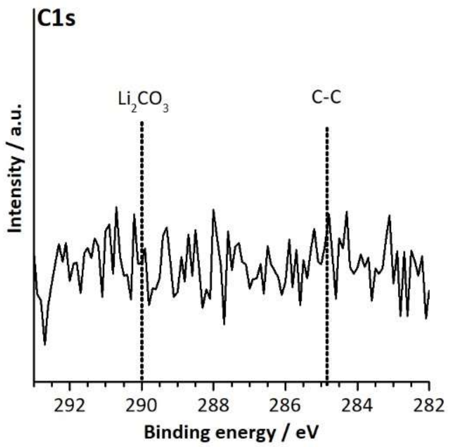

Figure S11. C1s detailed spectrum of the annealed LLZTO surface prior to the LiF interface experiment.
